# Supplementary material for: Content-rich biological network constructed by mining PubMed abstracts
Source: BMC Bioinformatics. 2004 Oct 8;5:147. doi: 10.1186/1471-2105-5-147 (PMC528731; doi:10.1186/1471-2105-5-147)
Supplement: Additional File 2 — The original results of the above study (non-essential files are deleted to keep the file size under the limit set by BMC bioinformatics). [file 1471-2105-5-147-S2.bz2 › chilibotAdditionalFile2/dip05/584ID9560191E10952/html/TNNC2_TNNI2.html]

 


 **TNNC2** and **TNNI2** 
  
Found 1 abstracts in PubMed, retrieved 1.  
 

 What does Google say? 
 PDF only 
| .edu only 

---

**Interactive relationship** (e.g. stimulation, inhibition, etc)

**Non-interactive relationship** (e.g. studied together, co-existance, homology, etc.)

- By radiation hybrid mapping, TPM1 was assigned to chromosome 15q22.1, TPM2 to chromosome 9p13.2 p13.1, TNNI1 to chromosome 1q31.3,  **TNNI2**  to chromosome 11p15.5, and  **TNNC2**  to chromosome 20q12 q13.11.  Ref: 9016781 Biochem Biophys Res Commun, 1997
- In this paper the chromosomal localization of the human skeletal muscle genes Troponin I slow twitch TNNI1, Troponin I fast twitch  **TNNI2** , and Troponin C fast  [ **TNNC2** ]   **TNNC2**  and the refinement of the position for alpha Tropomyosin TPM1 and beta Tropomyosin TPM2 are reported.  Ref: 9016781 Biochem Biophys Res Commun, 1997
